# Robust and automated three-dimensional segmentation of densely packed cell nuclei in different biological specimens with Lines-of-Sight decomposition

**DOI:** 10.1186/s12859-015-0617-x

**Published:** 2015-06-08

**Authors:** B. Mathew, A. Schmitz, S. Muñoz-Descalzo, N. Ansari, F. Pampaloni, E.H.K. Stelzer, S.C. Fischer

**Affiliations:** 1 Buchmann Institute for Molecular Life Sciences (BMLS), Fachbereich Biowissenschaften (FB15, IZN), Goethe Universität Frankfurt am Main, Max-von-Laue-Straße 15, 60438 Frankfurt am Main, Germany; 2 Department of Biology and Biochemistry, University of Bath, Bath, BA2 7AY UK

**Keywords:** Image segmentation, image processing, algorithm, approximately convex decomposition, clustering, three-dimensional microscopy, spheroid, mouse embryo

## Abstract

**Background:**

Due to the large amount of data produced by advanced microscopy, automated image analysis is crucial in modern biology. Most applications require reliable cell nuclei segmentation. However, in many biological specimens cell nuclei are densely packed and appear to touch one another in the images. Therefore, a major difficulty of three-dimensional cell nuclei segmentation is the decomposition of cell nuclei that apparently touch each other. Current methods are highly adapted to a certain biological specimen or a specific microscope. They do not ensure similarly accurate segmentation performance, i.e. their robustness for different datasets is not guaranteed. Hence, these methods require elaborate adjustments to each dataset.

**Results:**

We present an advanced three-dimensional cell nuclei segmentation algorithm that is accurate and robust. Our approach combines local adaptive pre-processing with decomposition based on Lines-of-Sight (LoS) to separate apparently touching cell nuclei into approximately convex parts. We demonstrate the superior performance of our algorithm using data from different specimens recorded with different microscopes. The three-dimensional images were recorded with confocal and light sheet-based fluorescence microscopes. The specimens are an early mouse embryo and two different cellular spheroids. We compared the segmentation accuracy of our algorithm with ground truth data for the test images and results from state-of-the-art methods. The analysis shows that our method is accurate throughout all test datasets (mean F-measure: 91 %) whereas the other methods each failed for at least one dataset (F-measure ≤ 69 %). Furthermore, nuclei volume measurements are improved for LoS decomposition. The state-of-the-art methods required laborious adjustments of parameter values to achieve these results. Our LoS algorithm did not require parameter value adjustments. The accurate performance was achieved with one fixed set of parameter values.

**Conclusion:**

We developed a novel and fully automated three-dimensional cell nuclei segmentation method incorporating LoS decomposition. LoS are easily accessible features that ensure correct splitting of apparently touching cell nuclei independent of their shape, size or intensity. Our method showed superior performance compared to state-of-the-art methods, performing accurately for a variety of test images. Hence, our LoS approach can be readily applied to quantitative evaluation in drug testing, developmental and cell biology.

**Electronic supplementary material:**

The online version of this article (doi:10.1186/s12859-015-0617-x) contains supplementary material, which is available to authorized users.

## Background

Biological processes rely on spatial cell-cell and cell-matrix interactions and are highly influenced by the microenvironment of the cells [1–3]. A large number of biomarkers and dyes are available to label distinct cellular structures. Well-defined protocols enable the observation and quantification of dynamic processes of cells within intrinsically three-dimensional structures. Several techniques have been explored to track cells *in vivo* or *in vitro*. The classical method is fluorescent labelling of cell nuclei [4, 5] and visualization by advanced three-dimensional fluorescence microscopy. This method enables the localization of cell nuclei and is also utilized to assess cell viability.

Optical imaging has experienced a significant progress during the past 20 years [6]. The most widely applied advanced three-dimensional fluorescence imaging technique is confocal microscopy [7, 8]. Light sheet-based fluorescence microscopy (LSFM) such as single plane illumination microscopy (SPIM) [9, 10] and digital scanned laser light sheet-based fluorescence microscopy (DSLM) ([11]) are becoming increasingly popular. They have demonstrated their applicability for imaging biological specimens ranging from a single cell to entire animals ([12, 13]). However, fluorescence images produced by all microscopes are affected by their optical properties. The brightness as well as the signal-to-noise ratio in images of large, thick and light-scattering multicellular specimens decreases with the penetration depth [14]. Furthermore, the axial resolution is at least three times worse than the lateral resolution, which often causes objects in the image to apparently touch one another.

The availability of three-dimensional microscopes for three-dimensional biological specimens has pushed the development of three-dimensional image segmentation methods. Three-dimensional cell nuclei segmentation methods commonly rely on pre-processing steps (e.g. filtering, initial thresholding or seed detection) combined with watershed ([15–17]), graph cut ([18, 19]), machine learning ([20, 21]), gradient flow tracking [22], active surface models [23], level set [24], or concavity-based segmentation ([25, 26]). But despite the continual progress in three-dimensional cell nuclei segmentation, there is still a need to improve accuracy, level of automation and adaptability.

A major challenge for segmentation is the separation of densely packed or apparently touching cell nuclei. Another difficulty arises from the diversity in terms of the imaged biological specimens or the imaging techniques used for acquisition. In general, segmentation methods are adopted to either a specific specimen (e.g. mouse embryo [8], *C. elegans* [27] or zebra fish [28]) or a specific imaging technique (e.g. DSLM or confocal microscope [29–31]); or their accuracy has only been tested for one particular application ([32, 33]). To achieve a satisfactory performance of such a method for images that contain another specimen or were obtained by a different imaging technique, sets of parameter values need to be optimized.

To tackle these problems and establish a method that does not rely on signal intensity, heavy parameterization or effective initialization of an algorithm, we chose approximate convex decomposition as the basis of our cell nuclei segmentation method. In the literature, several approaches exist for separating objects into approximately convex parts. However, none of them have been applied to biological images. In Lien’s and Amato’s approach [34], first the most concave regions are identified, and then they are partitioned such that the concavity is reduced below a specified threshold - resulting in approximately convex parts. Another approach is based on greedy region growing of a surface part until the distance to its convex hull decreases below a given threshold [27]. This again yields approximately convex parts. In Attene et al. [35], a shape is represented by a tetrahedral mesh. Decomposition is achieved by calculating a hierarchy of convex polyhedra that tightly enclose the shape. In a bottom-up manner, a single polyhedron is clustered into approximately convex parts.

In the work of Asafi *et al*. [36], approximate convex decomposition is achieved by analyzing pairs of surface points of a shape that are visible to each other. Lines between such mutually visible pairs of points are called “Lines-of-Sight” [36]. Neither a tetrahedralization nor the convex hull have to be calculated for an object. Therefore, Lines-of-Sight are easily accessible features for which neither shape and size information nor intensity distributions have to be considered. We apply this segmentation method to biological images for the very first time.

We combine the Lines-of-Sight (LoS) concept with a local adaptive pre-processing to separate apparently touching cell nuclei into approximately convex parts representing single cell nuclei. We show that our LoS implementation accurately segments three-dimensional cell nuclei due to effective separation of apparently touching cell nuclei. The accuracy of our proposed method exceeded 86 %. Furthermore, volumes of split cell nuclei were well represented. In a direct comparison with methods that are based on a graph cut algorithm (*FARsight*), seeded watershed transformation (ImageJ *3D Watershed*) or machine learning (*ilastik*) – commonly used techniques for three-dimensional cell nuclei segmentation – we demonstrate overall superior segmentation performance and volume measurements. Thereby, our automated LoS approach is applied with the same set of parameter values for real test images that differ in terms of biological specimen, size, imaging technique, and quality. Together, this demonstrates the high accuracy, adaptability and applicability of the proposed method.

## Method

### Approximately convex decomposition with Lines-of-Sight

**Fig. 1:**
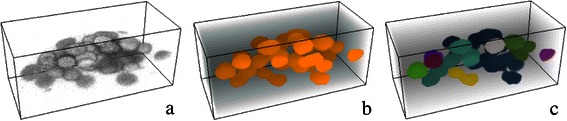
Densely packed and apparently touching cell nuclei challenge segmentation procedures. Illustration of segmentation challenges for a three-dimensional section of densely packed and apparently touching cell nuclei. (**a**) Section of a raw gray-scale image taken from a CD1 mouse embryo labelled with DAPI. (**b**) Cell nuclei after intensity thresholding. (**c**) Touching sets of nuclei are assigned identical colors, indicating that they are wrongly detected as on object

In many biological specimens cell nuclei are naturally densely packed. In three-dimensional fluorescence microscopy images, they even seem to touch each other, e.g. due to a lower axial than lateral resolution of the microscope. The differences in intensity are often not sufficient to distinguish single cell nuclei within a clump. Therefore, basic segmentation by intensity thresholding fails to identify individual nuclei (Fig. 1).

**Fig. 2:**
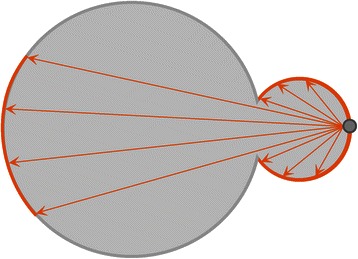
Concept of Lines-of-Sight (LoS). Lines-of-Sight are lines connecting two points on the surface of an object that do not leave the inner volume of the shape (orange lines). Pairs of surface points that are in line-of-sight are mutually visible. Consequently, the gray surface point is in line-of-sight with the orange marked regions

We developed an automated and robust three-dimensional cell nuclei segmentation. The idea is that a single nucleus is expected to be convex, whereas clumps of apparently touching cell nuclei are in general not convex. However, object decomposition into exact convex parts can be costly and too strict for segmentation due to the generation of an incontrollable number of components [34]. Hence, the concept of approximate convex decomposition was implemented to decompose apparently touching cell nuclei. The definition of approximately convex and the concept of Lines-of-Sight (LoS) was taken from the work of Asafi *et al*. [36] and was adapted to cell nuclei. Two surface points of a shape are said to be in line-of-sight if the connecting line between those two points does not leave the inner volume of the shape. Such mutually visible points are in a convex position. Hence, an object is convex if all surface points are in a convex position. If few surface points are not in a convex position, an object is approximately convex. Fig. 2 illustrates Lines-of-Sight for a given surface point. As evident, not all pairs of points that are in line-of-sight belong to a connected part of the shape surface. Thus, this object is not convex.

Using this definition, we cluster surface points that are in line-of-sight. To ensure robust clustering we determine an initial guess of the number of clusters. For a number of *k* clusters, each cluster represents a first approximation of an individual cell nucleus. Subsequently, this rough estimation is optimized such that we obtain clearly separated cell nuclei. The method was implemented as a processing pipeline for three-dimensional gray level images. The raw images pass through four main stages: (1) local adaptive binarization, (2) collecting connected components, (3) determining the number of divisible parts and (4) decomposition of components into approximately convex parts with LoS.

**Fig. 3:**
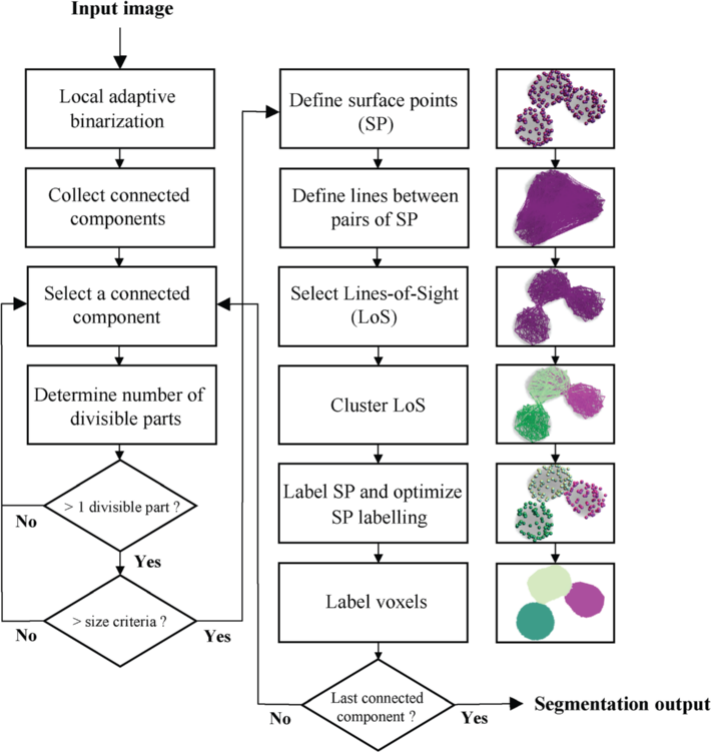
Steps of LoS decomposition algorithm for three-dimensional cell nuclei segmentation. Flow chart of the complete LoS decomposition pipeline. Pre-processing is depicted in the left column. The actual LoS decomposition steps (middle column) are visualized based on a clump of three apparently touching cell nuclei (right column). Colors indicate associated components in the example images. Touching cell nuclei appear in transparent gray

The details of the algorithm are described in the next section and illustrated in Fig. 3. Thereby, all parameter values described in the following section were determined by a parameter scan. We chose one common parameter set that showed equally good performance of our algorithm for all test datasets.

### Algorithm

#### Pre-processing

1. **Local adaptive binarization.** Foreground and background are separated by local intensity-based thresholding performed per slice. At each pixel, Otsu’s clustering method is applied to compute an intensity threshold within a defined neighborhood range of 18 pixels. Within this neighborhood, each pixel value above the local threshold is considered as foreground. This procedure returns an initial binary image, which might contain artefacts outside the region of interest (ROI). Therefore, a binary mask of the ROI is constructed by using a maximum filter with a large radius on the rough binarized raw image. Consequently, the ROI voxels are assigned a one and voxels outside the ROI a zero. Multiplication of the initial binary image with the ROI mask eliminates voxels outside the ROI. This yields an improved binary image. In a second step, holes within foreground objects are filled. Finally, a morphological opening removes objects that are smaller than a spherical structuring element of radius five. The resulting binary image provides the basis both for determining the number of divisible parts and for the decomposition of apparently touching nuclei.
2. **Collect connected components**. Connected foreground components of the given binary image are extracted and stored together with their bounding box specifying their exact location in the image. Each extracted foreground component corresponds to a possible clump of apparently touching cell nuclei and is individually processed by the LoS algorithm.
3. **Determine the number of divisible parts.** To determine the number of divisible parts for each connected component, we implemented an automatic detection method based on the Euclidean distance transformation. Thereby, the value of each voxel is replaced by its Euclidean distance to the closest background voxel. Consequently, the minimum distance from each voxel to the surface is returned. We determine the number of divisible parts by detecting local maxima in the transformed image that exceed the values of adjacent voxels by at least 0.1. For a convex object the local maximum is equivalent to the centroid. Objects that touch each other exhibit dents at split sites. Therefore, for a cell nuclei clump we detect one local maximum for each approximately convex part. Hence, the number of local maxima gives an approximation of the number of nuclei in a clump. An object with exactly one maximum represents a single cell nucleus.

#### LoS decomposition

The LoS algorithm is applied to each object that contains more than one local maximum and consists of less than 1000 voxels since both criteria give strong evidence of apparently touching cell nuclei. Locations of maxima are irrelevant and simply the number is considered for LoS decomposition. Each candidate object passes through seven steps for decomposition:

(i) **Define surface points.** All foreground voxels that are directly adjacent to a background voxel are set to be the surface points of an object.
(ii) **Define lines between pairs of surface points.** Pairs are formed from the determined surface points. To ensure good accuracy as well as fast processing, we perform a sampling. Thereby, the sample size of pairs depends on the number of surface points that are collected. We define a lower bound of 1000 surface points and an upper bound of 100000 surface points. If the number of surface points is below 1000, 10 % of the surface points are sampled. Exceeding 100000 surface points decreases the sample size to 2 %. If the number of surface points is in between the lower and upper bound, 3 % of the surface points are considered. Lines between pairs of surface points are generated.
(iii) **Select LoS**. To determine whether a line between two surface points defines a Line-of-Sight, we check whether the line leaves the object. Points are sampled along each line at an interval of 0.1 % of its length and for each sample point we check whether this point is part of the foreground. If this is true, such a line is considered to not leave the inner volume of the object and thus defines a Line-of-Sight. All other lines are not LoS and are discarded.
(iv) **Cluster LoS**. We perform a bottom-up hierarchical agglomerative clustering (HAC) of the LoS to subdivide them into *k* clusters, where *k* is given by the number of divisible parts determined in (3). The HAC algorithm always starts with each Line-of-Sight in one cluster. In each step, the similarity between two lines is computed with the chessboard distance and nearest clusters are fused using Ward’s method until *k* clusters are formed [37].
(v) **Label surface points**. Assuming the number of detected divisible parts for a nuclei clump is *k*, HAC outputs *k* clusters. LoS in the same cluster are assigned a common cluster label. LoS with different labels can originate from the same surface point. Therefore, the label of the surface point is set to the most common cluster label of the lines originating from that point. In case of an equal distribution of cluster labels, the cluster label is chosen randomly from the respective labels.
(vi) **Optimize surface points labelling**. To achieve a labelling of the surface points such that the points in the individual parts are geometrically connected, i.e. form a coherent labelled surface and therefore an individual object, an optimization of the previous surface points labelling is performed. Therefore, each surface point is assigned the most common surface point label of the eighteen nearest surface points.
(vii) **Label voxels**. Surface points labelling subdivides the surface of an object. To separate the whole object, we label the inner voxels according to the labelled surface points. For each foreground voxel the eighteen nearest surface points are determined. The voxel label is chosen as the most common label of these surface points. In case of an equal distribution of surface point labels, the label is determined randomly from the surface point labels found. The labelled voxels are then used to create a component matrix where each individual object obtains a unique identifier.

All previously described steps are performed for each nuclei clump. Subsequently, the already individual nuclei and the decomposed cell nuclei are reassembled and combined to a component matrix. Feature measurements such as the centroids and volumes of the objects are computed for each nucleus and stored in an Excel sheet.

#### Implementation

The method is implemented in *Mathematica* language (Version 9) and uses the *ImageJ* plugin for automatic local thresholding [38]. The source code is freely downloadable from [39]. Data processing using the complete pipeline with the test datasets took 8916 s for the mouse embryo, 3408 s for the breast cancer spheroid and 26487 s for the pancreatic cancer spheroid on a 2.67 GHz 12-core Intel Xeon E5650 with 96 GB of RAM. We expect to continue improving the performance of the LoS pipeline.

## Results

### Ethics statement

All mouse work was approved by the University of Bath Animal Welfare and Ethical Review Body (AWERB) and undertaken under UK Home Office license PPL 30/3219 in accordance with the Animals (Scientific Procedures) Act incorporating EU Directive 2010/63/EU. The human cell lines used in this study are listed in the *American Type Culture Collection* (ATCC) and therefore raise no ethical concerns (ATCC number for T47D: HTB-133 and for BxPC3: CRL-1687).

### Real test image data and ground truth data

**Fig. 4:**
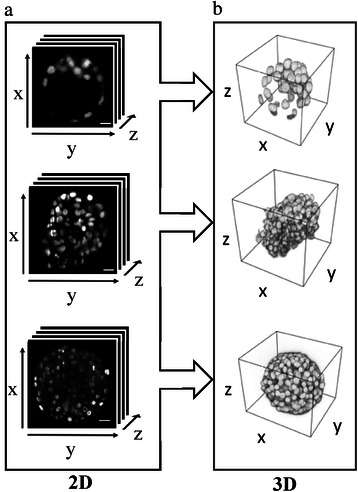
Cell nuclei in different biological specimens acquired with different imaging techniques. (**a**) Raw data showing single slices of three-dimensional stacks of three test datasets. From top to bottom: CD1 mouse embryo labelled with DAPI (scale bar 10 μm). In total 235 two-dimensional slices. Recorded with a Zeiss confocal microscope (LSM 780, AxioObserver), objective lens EC Plan-Neofluar 40x/1.30 Oil Ph3. Cellular spheroid of human breast cancer cells (T47D) labelled with H2B:GFP (scale bar 10 μm). In total 296 two-dimensional slices. Recorded with a digital scanned laser light sheet-based fluorescence microscope (DSLM), objective lens Epiplan-Neofluar 10x/0.3 NA. Large cellular spheroid of BxPC3 human pancreatic cancer cells labelled with DRAQ5 (scale bar 20 μm). In total 465 two-dimensional slices. Recorded with a selective plane illumination microscope (SPIM), objective lens CZ 40x/0.8 NA water dipping. (**b**) Three-dimensional image reconstruction of respective stacks of raw images

To validate our segmentation method, we quantified its performance with three-dimensional images of a mouse embryo recorded with a confocal microscope, a cellular spheroid of T47D human breast cancer cells recorded with a DSLM, and a large cellular spheroid of BxPC3 human pancreatic cancer cells recorded with a SPIM (Fig. 4). The mouse embryo is an example of a developmental system, whereas cellular spheroids are commonly used as tumur models. The images demonstrate various common challenges of cell nuclei segmentation: (1) varying intensities, (2) different types of cells of different sizes, (3) heterogeneous cell density and (4) an unpredictable degree of overlap. In addition, all images originate from different microscopes, which adds a level of technical variation. The file sizes of the three-dimensional stacks of images differ between 18.5 MB (512×512 pixels), 11.4 MB (285×284 pixels), and 203 MB (672×512 pixels) for the mouse embryo, the breast cancer spheroid, and the pancreatic spheroid, respectively. For each image, a ground truth (GT) dataset was generated by manual detection of the nuclei centroids.

### Comparison with state-of-the-art methods

The segmentation accuracy was not only compared with ground truth data, but also with results from the state-of-the-art segmentation methods *FARSight* ([40], [18]), *3D Watershed* in ImageJ ([41], [42]) and *ilastik* ([43],[20]). These are commonly used, open-source segmentation tools [6]. Additionally, we tested several other methods described in the literature, e.g. the method presented in [22], a three-dimensional cell nuclei segmentation named *“CellSegmentation3D”* based on gradient flow tracking and the software MINS [8]. All methods were supplied with the same raw images. A major drawback of many segmentation methods is their incapability to process huge datasets. MINS segmentation failed for the pancreatic spheroid and *“CellSegmentation3D”* as well as the command line tool for *FARSight* failed for all datasets, since they crashed during processing. Due to this issue with *FARSight’s* command line tool, we had to revert to the corresponding graphical user interface *“NucleusEditor”*. Furthermore, the segmentation tools do not support all image types. *“NucleusEditor”* e.g. did not operate with 16 bit images. Since the default parameter values of *FARSight* resulted in unsatisfactory segmentation results, we screened multiple sets of parameter values and chose the most reasonable one for each test image.

*3D Watershed* is a plugin for a seeded watershed within the 3D ImageJ suite. We used the automated seed detection implemented in this plugin. Further details are given in Additional file 1. Similar to *FARSight,* the parameter values had to be adjusted because the default values resulted in unsatisfactory segmentation results. For the machine learning-based segmentation tool *ilastik*, a training dataset had to be created for each test image. In contrast, the proposed LoS algorithm does not require a training dataset and uses the same parameter values for all test images.

### Accurate segmentation performance with LoS decomposition

**Fig. 5:**
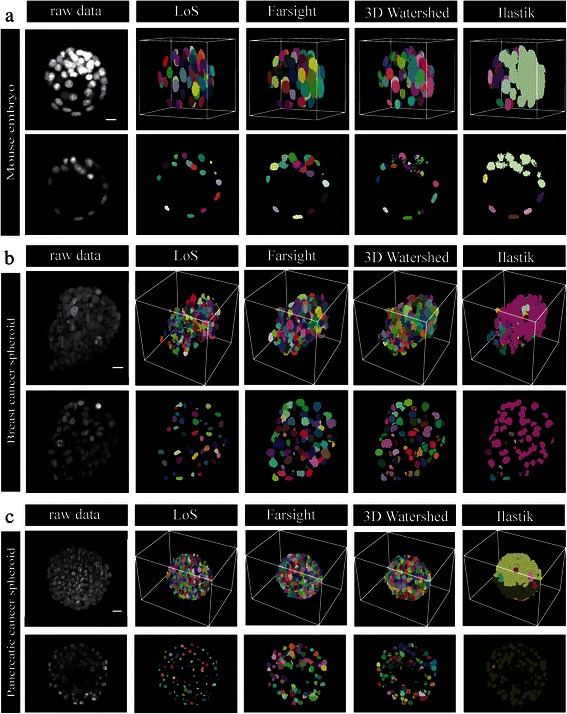
Comparison of LoS decomposition of the three test datasets with *FARSight*, *3D Watershed* and *ilastik*. Results for (**a**) the mouse embryo dataset, (**b**) the breast cancer spheroid dataset, and (**c**) the pancreatic cancer spheroid dataset. In each case, the top row shows the maximum projection of the raw three-dimensional data and the segmentation output generated by LoS, *FARsight*, *3D Watershed* and *ilastik*. Each segmented object is assigned a different color. The bottom row shows a two-dimensional section of the dataset (slice number for dataset (**a**): 123, (**b**): 146, and (**c**): 292) and the corresponding sections of the segmentations. Scale bars: (**a**) 10 μm, (**b**) 20 μm, (**c**) 20 μm

**Fig. 6:**
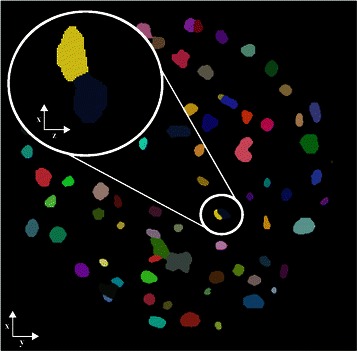
Results of LoS decomposition along XY and XZ. Visualization of one two-dimensional slice along XY through the three-dimensional stack of the pancreatic cancer spheroid after LoS decomposition. The smaller inset shows the separated cell nuclei in XY and the bigger inset shows the magnification of the result in XZ

**Fig. 7:**
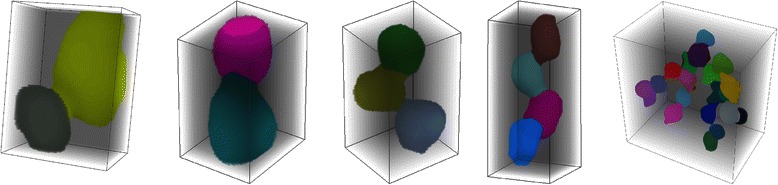
Examples of separated clumps of apparently touching cell nuclei. Each part within a cell nuclei clump is assigned a different color

Fig. 5 shows the segmentation results achieved by our LoS implementation, *FARSight*, *3D Watershed* and *ilastik* in three dimensions and in a single two-dimensional slice. Additional file 2: Figure S2) shows further details of segmentation results along XZ and YZ. *Ilastik* failed for all datasets. *3D Watershed* and *FARSight* suffered from over-segmentation (Fig. 5a and c and Additional file 2: Figure S2C). Furthermore, *3D Watershed* segmentation resulted in nearly straight borders between cell nuclei (Fig. 5b and Additional file 2: Figure S2B). For all other methods the shapes of the splitting sites seem more natural. Note that cell nuclei that appear split incorrectly in the depicted two-dimensional slices are found to be correctly split if one considers the XZ direction (Fig. 6). Fig. 7 shows three-dimensional renderings of single clumps of apparently touching cell nuclei after decomposition with LoS. Clumps with nuclei of different sizes or shapes are similarly well split as clumps of homogenously sized or shaped nuclei. Furthermore, small and large clumps are split equally well.

For a quantitative analysis of the segmentation results of LoS and the other methods, four well-established metrics were used ([32, 44]):

$$ \mathrm{R}\mathrm{e}\mathrm{call}=\frac{\mathrm{TP}}{\mathrm{TP}+\mathrm{F}\mathrm{N}} $$

$$ \Pr \mathrm{ecision}=\frac{\mathrm{TP}}{\mathrm{TP}+\mathrm{F}\mathrm{P}} $$

$$ \mathrm{F}\hbox{-} \mathrm{measure}=2\frac{\mathrm{Precision}\times \mathrm{Recall}}{\mathrm{Precision}+\mathrm{Recall}} $$

$$ \mathrm{Accuracy}=\frac{\mathrm{TP}}{\mathrm{TP}+\mathrm{F}\mathrm{N}+\mathrm{F}\mathrm{P}} $$

For each segmentation method true positive (TP), false positive (FP) and false negative (FN) represent the number of correctly detected, falsely detected, and undetected nuclei relative to the GT [32].

To calculate these four metrics, we matched the centroids determined by the segmentation to centroids found in the GT. To do this, a three-dimensional spherical neighborhood of a range that corresponds to the average diameter of the respective cell nuclei was centered at each centroid position in the GT. If this local neighborhood included exactly one centroid in the segmentation, we counted it as a correct detection – thus true positive (TP). If the neighborhood included more than one centroid, the closest one was considered as TP. These calculations resulted in a TP score for each three-dimensional test image. Based on the number of TP, scores for false positive (FP) and false negative (FN) were obtained using FP = n_seg_ - TP and FN = n_gt_ - TP, where n_seg_ and n_gt_ are the number of centroids determined by the segmentation and centroids in the GT for each dataset [32].

Based on these classifications, we measured precision, recall, accuracy, and F-measure of our LoS method and the state-of-the-art methods. Table 1 shows the performance of LoS, *FARSight*, *3D Watershed*, and *ilastik* for all three test images. We observed that for recall all methods achieved comparable results. Hence, the number of detected nuclei for each method was similar to the number of cell nuclei in the GT. Thus, there was not much under-segmentation. However, compared to the LoS approach, all other methods exhibit lower precision. Specifically, we observed that single cell nuclei are mistakenly split into multiple parts, increasing the over-segmentation rate. The performance of the LoS algorithm was steadily accurate for the different datasets. In the worst case, we obtained an F-measure of 86 %, whereas the lowest F-measure for the other methods was 69 %, 34 %, and 5 % for *FARSight*, *3D Watershed*, and *ilastik*, respectively (see Table 1).

**Table 1:** Algorithmic performance of LoS, *FARSight*, *3D Watershed* and *ilastik*

Dataset	# cells GT	Algorithm	# cells Seg	Match	Recall	Precision	Accuracy	F-measure
**Mouse embryo**	61	LoS	59	58	0.95	0.98	0.94	0.97
		FARSight	62	60	0.98	0.97	0.95	0.98
		3D Watershed	299	61	1.00	0.20	0.20	0.34
		ilastik	274	61	1.00	0.22	0.22	0.36
**Breast cancer spheroid**	240	LoS	247	216	0.90	0.87	0.80	0.89
		FARSight	338	236	0.98	0.70	0.69	0.82
		3D Watershed	288	220	0.92	0.76	0.71	0.83
		ilastik	112	74	0.31	0.66	0.27	0.42
**Pancreatic cancer spheroid**	531	LoS	690	523	0.98	0.76	0.75	0.86
		FARSight	997	524	0.99	0.53	0.52	0.69
		3D Watershed	734	518	0.98	0.70	0.69	0.81
		ilastik	19744	531	1.00	0.03	0.03	0.05

Performance was measured against manually segmented ground truth for the three different test datasets. “# cells GT”, “# cells Seg” and “Match” list the number of cells that were determined manually in the ground truth, segmented by the different algorithms, and matched, respectively. The segmentation performance is given in terms of the metrics “Recall”, “Precision”, “Accuracy” and “F-measure”. Thereby, values range from 0 (worst performance) to 1 (best performance). For the LoS algorithm the same set of parameter values was used for all test images. For *3D Watershed* and *FARSight* different parameter sets had to be used. These were determined by parameter scanning

These results clearly demonstrate that our proposed segmentation method is capable of achieving a consistent segmentation performance for diverse datasets compared to an ideal ground truth. Additionally, no adjustments of parameter values are required, thus making the method robust for these datasets and straightforward to use.

To investigate the contribution of our pre-processing steps and the LoS decomposition to the performance of the whole LoS pipeline, we performed a more detailed comparison with *3D Watershed.* We considered two additional cases for *3D Watershed*: (1) we used the binary image generated by the LoS pipeline and applied the distance transformation implemented in ImageJ and (2) we supplied the binary image and the seeds both generated by the LoS pipeline. Comparison showed that the nuclear decomposition capabilities are similar (see Additional file 1: Table S1). Using either the binary image generated by the LoS pipeline as input or the local maxima from our detection of divisible parts as seeds lead to an improvement of the performance of *3D Watershed*. Although MINS crashed for the pancreatic spheroid, we analyzed the performance for the two other datasets. Results are summarized in Additional file 3: Table S2 shows similar performance for LoS and MINS.

### Reliable nuclear volume representation with LoS decomposition

**Fig. 8:**
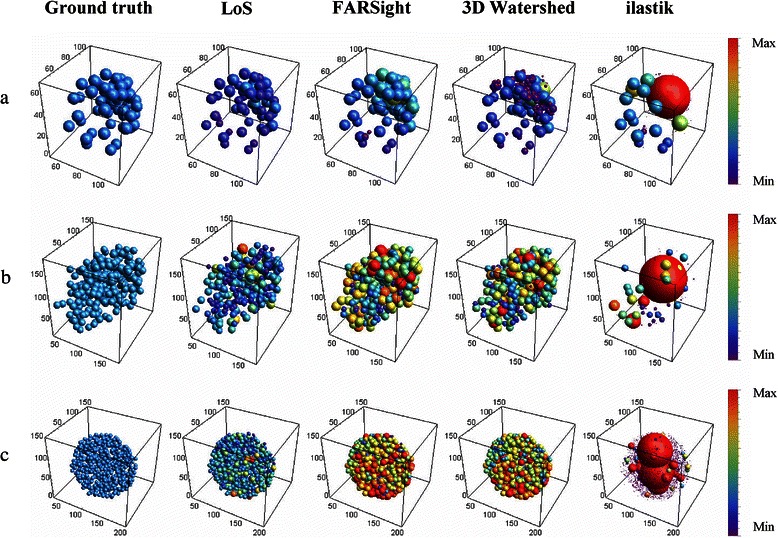
Visual comparison of nuclei volumes measured in segmentation results of LoS, *FARSight*, *3D Watershed* and *ilastik*. (**a**) Mouse embryo, (**b**) breast cancer spheroid and (**c**) pancreatic cancer spheroid. Spheres were plotted around each nucleus’ centroid position detected in the respective ground truth or by the respective segmentation method. The radius of each sphere in the ground truth represents the manually determined mean nuclei volume for a representative subset of nuclei. The spheres for the segmentation outputs represent the automatically determined volume for each detected nucleus. Coloring scheme: for each dataset, a minimum (purple) and an approximate maximum volume (red) across all results for LoS, *FARsight*, *3D Watershed* and *ilastik* was determined (minimum volume for coloring: 1 μm^3^ for (**a**), (**b**) and (**c**); maximum volume for coloring: 1600 μm^3^ for (**a**), 4200 μm^3^ for (**b**) and 1000 μm^3^ for (**c**), respectively). Note: for better visualization, extreme outliers from the *ilastik* segmentation results were removed for determining the maximum volume: (one for (**a**), one for (**b**) and three for (**c**)). Subsequently, each sphere was assigned a color depending on its volume

**Fig. 9:**
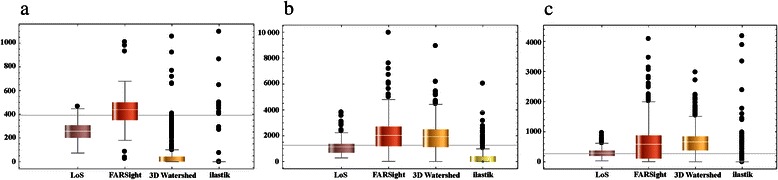
Quantitative comparison of nuclei volumes measured in segmentation results of LoS, *FARSight*, *3D Watershed* and *ilastik.* (**a**) Mouse embryo, (**b**) breast cancer spheroid and (**c**) pancreatic cancer spheroid. Data is visualized as box-and-whisker plots: the white lines within the boxes describe the respective mean, the error bars show variability outside the upper and lower quartiles and the dots represent outliers. The gray vertical line describes the mean of the respective ground truth nuclear volume that was manually determined for a subset of nuclei in all test datasets. Note: for better visualization extreme outliers from the segmentation results of *ilastik* were removed (one for (**a**), one for (**b**) and three for (**c**))

The volume of an individual nucleus is an important feature for the quantitative analysis of various processes. E.g. in drug screening assays the efficacy of a drug is expected to correspond to a decrease in nuclear volume. Hence, reliable representation of nuclear volumes after segmentation is essential. Therefore, we measured the nuclear volumes obtained by each segmentation method and compared it with a ground truth (GT) volume. The GT volume was manually determined based on the mean volume of representative 10 % of the cell nuclei from different regions of the respective test datasets (mouse embryo: 391 ± 94 μm^3^ (*n* = 6), breast cancer spheroid: 1260 ± 426 μm^3^ (*n* = 20), pancreatic cancer spheroid: 271 ± 102 μm^3^ (*n* = 50)). We found that for the mouse embryo, the mean nuclear volume achieved by *FARSight* segmentation was closer to the mean GT volume than the mean nuclear volume after LoS decomposition (Fig. 8a). However, after the LoS approach the nuclear volumes were more consistent with the ground truth than the result from *FARSight* as indicated by a smaller standard deviation (Fig. 9a; LoS: 257 ± 86 μm^3^ (*n* = 59), *FARSight*: 439 ± 180 μm^3^ (*n* = 62)). We observed, that compared to the standard deviation of the GT, the standard deviation of LoS was increased by 20 %. The other methods showed an increase in the standard deviation by 54 % for *FARSight*, 189 % for *3D Watershed*, and 8118 % for *ilastik*.

For the spheroids, the nuclear volumes from our LoS algorithm reproduced the true nuclear volumes better than all other three methods (Figs. 8b and c, 9b and c). *FARSight* and *3D Watershed* produced enlarged nuclei compared to the GT. *Ilastik* throughout failed to resemble the volumes for all datasets due to its lacking capability of separating apparently touching cell nuclei. To show that our LoS algorithm yields a significant improvement over the other methods, a Wilcoxon rank sum test with Holm’s correction was performed. Thereby, nuclear volumes achieved by LoS were always significantly different from those determined by all other methods (*p*-value < 0.01).

In summary, our results show that nuclear volumes obtained by the LoS method are more consistent with the ground truths than all other tested methods. Additionally, less outliers indicate the robustness of the method. These results demonstrate that LoS decomposition yields nuclear volumes that resemble the actual volumes very closely.

## Discussion

We developed a segmentation pipeline for cell nuclei in three-dimensional fluorescence images. The algorithm consists of local adaptive pre-processing followed by decomposition into approximately convex objects. Our detailed analysis revealed the contributions of the two parts to the robustness of the algorithm with respect to differences in image properties like the biological specimen, imaging technique, signal quality, intensity distribution and size of the cell nuclei. These major parts are discussed in the following sections.

## Pre-processing

The key steps in our pre-processing are a local adaptive binarization and the determination of the number of divisible parts.

During binarization, many regions are difficult to classify as foreground or background in images that exhibit background noise or differences in contrast and intensity. For such cases, local thresholding is more appropriate and robust than global thresholding. Applying Otsu’s clustering method locally yielded fewer clumped nuclei than global thresholding. Additionally, for the chosen binarization method only a single parameter value had to be adjusted: the radius that defines the neighborhood during local Otsu thresholding. A value for the radius that is too high can increase the number of apparently touching nuclei and decrease the total number of extracted cell nuclei. We found that a value that corresponds to the approximate radius of a cell nucleus yielded the best results. It is an intuitive feature of the biological specimen. In contrast, pre-processing implemented in e.g. *3D Watershed* involves non-intuitive adjustments of parameter values like an intensity threshold for the image (see Additional file 1: Figure S1). Defining intensity thresholds is typically image dependent and therefore sensitive to the signal-to-noise ratio of the image [22]. Such parameters need to be carefully set to avoid poor segmentation results [45]. Besides the easy and intuitive parameterization for this step, our approach is insensitive to non-uniform intensity distributions as well as the decreasing signal quality with increasing penetration depth into the specimen (Fig. 4). Differences in terms of image origin and image quality is compensated by this. Summarizing the above, this part of the pre-processing is robust if cell nuclei sizes do not differ excessively. Thus, we obtained good results for all our test datasets. Further improvements are possible. By changing the parameter value for the local neighborhood radius e.g. for the pancreatic cancer spheroid to 30 voxels, yields a segmentation improvement of about 7 % (F-measure 93 %) compared to the result shown in Table 1.

The determination of the number of divisible parts of an object can be responsible for over- and under-segmentation. A small value for the local maxima detection in the distance transformed image increases the number of local maxima and hence the number of potential cell nuclei. We found our value gives an accurate and robust initial guess of the number of divisible parts. In our algorithm, the number of divisible parts determines the number of LoS clusters. However, it is only an upper threshold for the number of segmented cell nuclei due to the steps that follow the LoS clustering. These ensure an optimized number of cell nuclei in the final output. If the cell nuclei in the biological specimen exhibit a homogeneous size distribution, an improvement of the detection of divisible parts may be possible by adding an optimization that considers reasonable size descriptors. In this case, huge cell nuclei clumps for which not enough divisible parts are detected, would be split into parts of an appropriate size.

Consequently, our implementation of the intuitively parameterizable binarization and the calculation of the number of divisible parts with the potential of a subsequent optimization, contribute to the robustness of our approach.

### LoS decomposition

The fundamental difference between our decomposition method and existing methods is the application of lines that define approximate convex parts. The robustness is achieved because LoS are easily accessible features of cell nuclei clumps that do not require previous knowledge about object shape, size or intensity. Hence, a good throughout performance of the decomposition of cell nuclei for all datasets was obtained with the same set of parameter values.

Clumps containing nuclei of different sizes or shapes are equally well split as clumps consisting of equally sized or shaped parts (Fig. 7). This is especially advantageous in the case of homeostatic tissues where the stem cell nuclei are in general smaller than the differentiated cell nuclei. Furthermore, for separating complex clumps of many nuclei our LoS algorithm exhibits a better performance than e.g. *3D Watershed*, *ilastik* or methods used in [46] or [25] (Fig. 7, most right).

One reason for the robustness is, that the decomposition is performed directly on the three-dimensional objects rather than applying it on each two-dimensional slice independently like in [47] or [19]. Incorporating the three-dimensional information provides more accurate decomposition of apparently touching cell nuclei. Assuming we would have considered the slice in Fig. 6 independently, the object that is depicted in the smaller inset would not have been separated since it is already approximately convex. However, the view along the XZ direction in the larger inset clearly shows that this object had to be split. Although here, we focus on three spatial dimensions, the method works well for two-dimensional images and has the potential of being extended to three-dimensional time-lapse images.

If the split site of an object is pronounced, a reliable LoS clustering is guaranteed. Clumps with small concavities (i.e. wide split sites) are challenging. In this case, more LoS can pass through the split site and cause mis-clustering of the lines. To overcome this effect, we have introduced a parameter for the sampling size of LoS and the steps “label surface points and optimize surface points labelling” that follow the LoS clustering. The number of sampled LoS determines the spacing between the lines within an object. Lines with a small distance between them are clustered together with a higher probability. The more lines are sampled, the more pass through the wide split site and thus are clustered together. Consequently, surface points of one part of the object are hit more often by LoS with different labels. This can lead to a wrongly labelled surface point. If this happens for too many surface points, it is hard to eliminate them even with the steps following the clustering. However, too few lines would not cover the object and therefore would not be sufficient for an accurate clustering. Therefore, we introduced a LoS sample size that changes if the number of surface points drops below a lower threshold or exceeds an upper threshold. Nonetheless, the algorithm is less sensitive to the values for the lower and upper threshold than to the LoS sample size. For all three parameters, we chose values that were equally applicable to the various test images.

In general, the surface points labelling works well since a surface point is hit more often by LoS with the correct label. Optimization of the surface points labelling eliminates the few remaining mis-labelled surface points that are surrounded by correctly labelled surface points.

To sum up, the surface points labelling and the optimization following the LoS clustering improve the decomposition performance. The sample size of the lines is one key parameter for the LoS decomposition which can affect the segmentation output. This value can be adapted to further improve the results. In general, we found that our value enables a robust decomposition for all our test images.

### Complete LoS pipeline

Applying the entire pipeline as depicted in Fig. 3 on the different test datasets revealed a throughout robust performance with the same set of parameter values. In contrast, the other methods were supplied with adjusted parameter values for each image. Although each applied method failed for at least one dataset, *FARSight* and LoS achieved similar results for the mouse embryo (see Table 1). Nevertheless, cell nuclei volume measurements of our LoS algorithm are more consistent with the manually obtained volumes than those determined by *FARSight*. This shows that the segmentation with *FARSight* worked accurately for the mouse embryo but it produced a cell nuclei volume distribution that did not reflect the ground truth volume.

For *3D Watershed* pre-processing can be decoupled from the cell nuclei decomposition step. Improved results of watershed segmentation with parts of our pre-processing reveal that our pre-processing steps are more robust for our test datasets than those implemented in the *3D Watershed* plugin (see Table S1 in Additional file 1). Moreover, for *3D Watershed* the number of seeds and the number of cell nuclei in the output are exactly the same. This requires a more accurate seed detection as is needed for our LoS approach. Our end result does not only rely on the initial number of divisible parts. The additional optimization steps after LoS clustering decouple the final result from the initial calculation, and thereby contribute to the robustness and accuracy of our algorithm.

In our pipeline, we discarded segmented cell nuclei below a defined volume threshold, a post- processing step that is applicable to a wide range of biological specimens. However, our segmentation method extracts further properties such as the centroids or the fluorescence intensity of the cell nuclei. If only a specific biological specimen is considered, this information can be used for more sophisticated post-processing steps.

## Conclusion

The automated, robust and accurate detection of a single cell nucleus is a crucial and challenging step for a precise quantification in many biological applications. Up to now, several three-dimensional cell nuclei segmentation methods exist but in most cases they need further adjustments to the considered image data and biological specimen. We introduce a three-dimensional segmentation method that incorporates separating apparently touching cell nuclei. It combines local adaptive pre-processing with a Lines-of-Sight approach for approximately convex decomposition. Our method is readily applicable to three-dimensional microscopy images of different biological specimens acquired by diverse imaging techniques.

The segmentation quality is largely independent of cell type, size, intensity, cell density, and image quality. A comparison with state-of-the-art algorithms revealed an overall better performance of our method in terms of identification of individual cell nuclei and extraction of relevant features such as the nuclear volume.

Three-dimensional cell nuclei segmentation with our LoS method provides the crucial starting point for many applications. Cellular spheroids e.g. serve as in vitro tissue models, especially in tumur biology. They show an improved resemblance of the spatial arrangements compared to two-dimensional cell cultures [48]. Therefore, they have become increasingly important for drug testing assays. Volume changes of cell nuclei within such spheroids upon drug treatment might provide insights into drug efficacy. Developmental systems such as mouse embryos serve as a good model for exploring the coordination of cell linage specification and morphogenesis [49]. Thereby, a reliable identification of cell nuclei positions facilitates accurate tracking and lineaging of cell nuclei during different processes and hence opens up a variety of possibilities for investigating cellular dynamics. In general, the wide applicability of our proposed segmentation method enables a reliable and robust quantification of cell nuclei in fluorescence images that will contribute to our knowledge in many biological applications.

## Additional files

Additional file 1:
***3D Watershed*** **with different pre-processing steps.** Comparison of the performance of our LoS algorithm with *3D Watershed* combined with different pre-processing steps.
Additional file 2: Figure S2. Visualization of different segmentation results along the XZ- and YZ-direction. Results for (A) the mouse embryo dataset, (B) the breast cancer spheroid dataset, and (C) the pancreatic cancer spheroid dataset. In each case, the top row shows two-dimensional sections of the raw image and the segmentation results along XZ direction. The bottom row shows two-dimensional sections of the raw image and the segmentation results along YZ direction. For each dataset, the methods with the best and second best F-measure are compared. Scale bars: (A) 10 μm, (B) 20 μm, (C) 20 μm.
Additional file 3:
**Comparison of LoS with MINS.** Segmentation performance of LoS and MINS.
