# Supplementary material for: Robust and automated three-dimensional segmentation of densely packed cell nuclei in different biological specimens with Lines-of-Sight decomposition
Source: BMC Bioinformatics. 2015 Jun 8;16:187. doi: 10.1186/s12859-015-0617-x (PMC4458345; doi:10.1186/s12859-015-0617-x)
Supplement: Additional file 1: — 3D Watershed with different pre-processing steps. Comparison of the performance of our LoS algorithm with 3D Watershed combined with different pre-processing steps. [file 12859_2015_617_MOESM1_ESM.pdf]

Additional file 1

Robust and automated three-dimensional segmentation of densely packed cell nuclei in different biological specimens with Lines-of-Sight decomposition

B. Mathew[1*], A. Schmitz[1], S. Muñoz-Descalzo[2], N. Ansari[1], F. Pampaloni[1], E.H.K. Stelzer[1], S.C. Fischer[1]

To compare the decomposition performance of the seeded ImageJ *3D Watershed* with our LoS approach, we applied *3D Watershed* with different pre-processing steps.

In general, three parameters can be adjusted for the ImageJ *3D Watershed* plugin (Figure S1). The parameter "Seeds Threshold" only considers seeds with a value larger than "Seeds Threshold". "Image Threshold" is used to cluster voxels with values larger than "Image Threshold". If automatic seed detection is chosen, the parameter "Radius for automatic seeds" can be set accordingly. These parameters only affect the pre-processing steps prior to the watershed decomposition. We chose three cases:

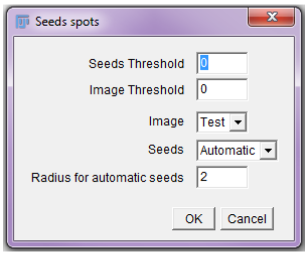

Figure S1. Possible parameter value adjustment for the *3D Watershed* plugin.

3D Watershed I. The raw image was supplied as input. The image threshold was set according to the respective image (40 for the mouse embryo, 500 for the breast cancer spheroid and 33100 for the pancreatic cancer spheroid). The automated seed detection was used with an appropriate radius for each test image (18 for the mouse embryo, 10 for the breast cancer spheroid and 15 for the pancreatic cancer spheroid). The results are displayed in Table 1 in the main text.

3D Watershed II. We used the plugin as described in "Example 1" (see http://imagejdocu.tudor.lu/doku.php?id=plugin:segmentation:3d_spots_segmentation:start). Thereby, we used the binary image generated in the binarization step of our LoS pipeline as

input. A 3D distance map (ImageJ plugin: “3D Distance Map”) was generated to get a distance transformed image as pre-step for the automated seed detection. An appropriate radius for the automatic seed detection was chosen (18 for the mouse embryo, 10 for the breast cancer spheroid and 15 for the pancreatic cancer spheroid).

***3D Watershed* III**. We supplied the binary image generated in the binarization step of our LoS pipeline as input. Additionally, we provided the local maxima derived for the determination of divisible parts as seeds. No further adjustments were made.

Table S1 shows the segmentation results with different pre-processing steps for *3D Watershed* compared with LoS. *3D Watershed II* and *III* adaptions achieve similar results as LoS for all test images. Especially *3D Watershed III* shows the most improvement compared to the other two watershed variations.

Table S1. Decomposition performance of LoS and variations of *3D Watershed.*

Dataset	# cells GT	Algorithm	#cells Seg	Match	Recall	Precision	Accuracy	F-measure
**Mouse embryo**	61	**LoS**	**59**	**58**	**0.95**	**0.98**	**0.94**	**0.97**
		3D Watershed I	299	61	1.00	0.20	0.20	0.34
		3D Watershed II	70	60	0.98	0.86	0.85	0.92
		3D Watershed III	63	60	0.98	0.95	0.94	0.97
**Breast cancer spheroid**	240	**LoS**	**247**	**216**	**0.90**	**0.87**	**0.80**	**0.89**
		3D Watershed I	288	220	0.92	0.76	0.71	0.83
		3D Watershed II	322	230	0.96	0.71	0.69	0.82
		3D Watershed III	278	228	0.95	0.82	0.79	0.88
**Pancreatic cancer spheroid**	531	**LoS**	**690**	**523**	**0.98**	**0.76**	**0.75**	**0.86**
		3D Watershed I	734	518	0.98	0.70	0.69	0.81
		3D Watershed II	804	529	1.00	0.66	0.66	0.79
		3D Watershed III	764	528	0.99	0.69	0.69	0.82

Performance was measured against manually segmented ground truth for the three different test datasets. “# cells GT”, “# cells Seg” and “Match” list the number of cells that were determined manually in the ground truth, segmented by the different algorithms, and matched, respectively.

The segmentation performance is given in terms of the metrics “Recall”, “Precision”, “Accuracy” and “F-measure”. Thereby, values range from 0 (worst performance) to 1 (best performance). Results for LoS were achieved as described in the main text (compare Table 1).
