# Supplementary figures and images for: Robust and automated three-dimensional segmentation of densely packed cell nuclei in different biological specimens with Lines-of-Sight decomposition
Source: BMC Bioinformatics. 2015 Jun 8;16:187. doi: 10.1186/s12859-015-0617-x (PMC4458345; doi:10.1186/s12859-015-0617-x)

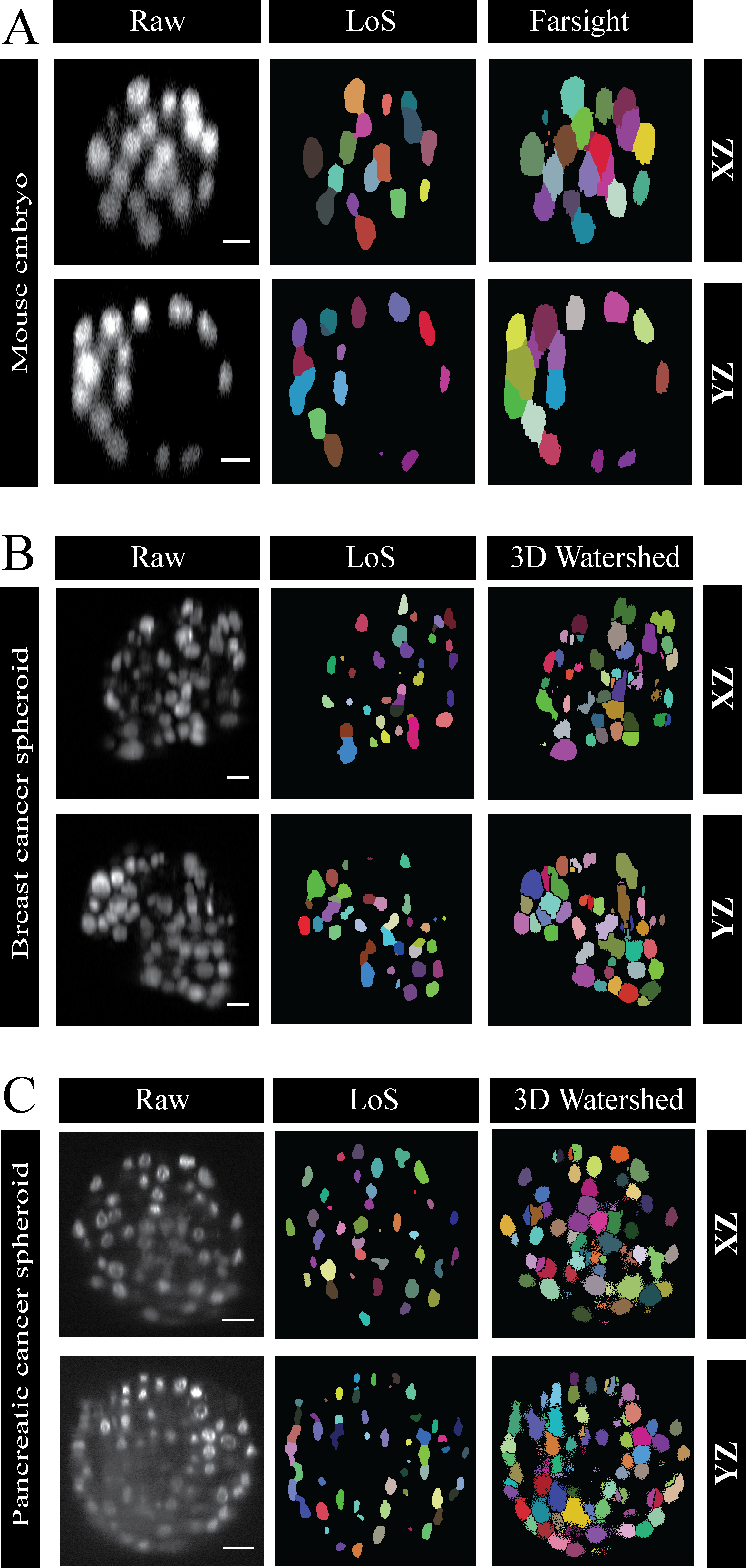

Supplement: Additional file 2: Figure S2. — Visualization of different segmentation results along the XZ- and YZ-direction. Results for (A) the mouse embryo dataset, (B) the breast cancer spheroid dataset, and (C) the pancreatic cancer spheroid dataset. In each case, the top row shows two-dimensional sections of the raw image and the segmentation results along XZ direction. The bottom row shows two-dimensional sections of the raw image and the segmentation results along YZ direction. For each dataset, the methods with the best and second best F-measure are compared. Scale bars: (A) 10 μm, (B) 20 μm, (C) 20 μm. [file 12859_2015_617_MOESM2_ESM.tiff]
