# Supplementary material for: Robust and automated three-dimensional segmentation of densely packed cell nuclei in different biological specimens with Lines-of-Sight decomposition
Source: BMC Bioinformatics. 2015 Jun 8;16:187. doi: 10.1186/s12859-015-0617-x (PMC4458345; doi:10.1186/s12859-015-0617-x)
Supplement: Additional file 3: — Comparison of LoS with MINS. Segmentation performance of LoS and MINS. [file 12859_2015_617_MOESM3_ESM.pdf]

Additional file 2

Robust and automated three-dimensional segmentation of densely packed cell nuclei in different biological specimens with Lines-of-Sight decomposition

B. Mathew[1*], A. Schmitz[1], S. Muñoz-Descalzo[2], N. Ansari[1], F. Pampaloni[1], E.H.K. Stelzer[1], S.C. Fischer[1]

The nuclear segmentation tool MINS was used according to the descriptions given in the supplementary material of Lou *et* al.: A rapid and efficient 2D/3D nuclear segmentation method for analysis of early mouse embryo and stem cell image data. Stem Cell Reports 2014.

Table S2 shows the comparison of the segmentation performance between LoS and MINS. MINS achieved similar results as LoS for the mouse embryo dataset and the breast cancer spheroid dataset. For the pancreatic cancer spheroid dataset MINS crashed.

Table S2. Segmentation performance of LoS and MINS.

Dataset	# cells GT	Algorithm	#cells Seg	Match	Recall	Precision	Accuracy	F-measure
**Mouse embryo**	61	**LoS**	**59**	**58**	**0.95**	**0.98**	**0.94**	**0.97**
		MINS	55	54	0.89	0.98	0.87	0.93
**Breast cancer spheroid**	240	**LoS**	**247**	**216**	**0.90**	**0.87**	**0.80**	**0.89**
		MINS	301	233	0.97	0.77	0.76	0.86
**Pancreatic cancer spheroid**	531	**LoS**	**690**	**523**	**0.98**	**0.76**	**0.75**	**0.86**
		MINS	NA	NA	NA	NA	NA	NA

Performance was measured against manually segmented ground truth for the three different test datasets. “# cells GT”, “# cells Seg” and “Match” list the number of cells that were determined manually in the ground truth, segmented by the different algorithms, and matched, respectively. The segmentation performance is given in terms of the metrics “Recall”, “Precision”,

“Accuracy” and “F-measure”. Thereby, values range from 0 (worst performance) to 1 (best performance). Results for LoS were achieved as described in the main text (compare Table 1). MINS crashed for the pancreatic cancer spheroid, thus no results are available. For the other two test images different parameter sets had to be used. These were determined by parameter scanning.
